# Selenium Derivatives as Promising Therapy for Chagas Disease: *In Vitro* and *In Vivo* Studies

**DOI:** 10.1021/acsinfecdis.1c00048

**Published:** 2021-04-19

**Authors:** Rubén Martín-Escolano, Mikel Etxebeste-Mitxeltorena, Javier Martín-Escolano, Daniel Plano, María J. Rosales, Socorro Espuelas, Esther Moreno, Manuel Sánchez-Moreno, Carmen Sanmartín, Clotilde Marín

**Affiliations:** † Laboratory of Molecular & Evolutionary Parasitology, RAPID group, School of Biosciences, University of Kent, Canterbury CT2 7NJ, United Kingdom; ‡ Facultad de Farmacia y Nutrición, Departamento de Tecnología y Química Farmacéuticas, Universidad de Navarra, Irunlarrea, 1, E-31008 Pamplona, Spain; § Instituto de Salud Tropical, Universidad de Navarra (ISTUN), Irunlarrea, 1, E-31008 Pamplona, Spain; ∥ Instituto de Investigaciones Sanitarias de Navarra (IdiSNA), Irunlarrea, 1, E-31008 Pamplona, Spain; ⊥ Department of Parasitology, Instituto de Investigación Biosanitaria (ibs. Granada), Hospitales Universitarios De Granada/University of Granada, Severo Ochoa s/n, 18071 Granada, Spain; ¶ Servicio de Microbiologia Clinica y Enfermedades Infecciosas, Hospital General Universitario Gregorio Marañón, 28007 Madrid, Spain; △ Instituto de Investigación, Sanitaria Gregorio Marañón (IiSGM), 28009 Madrid, Spain

**Keywords:** Chagas disease, chemotherapy, drug discovery, selenium derivatives, *Trypanosoma cruzi*

## Abstract

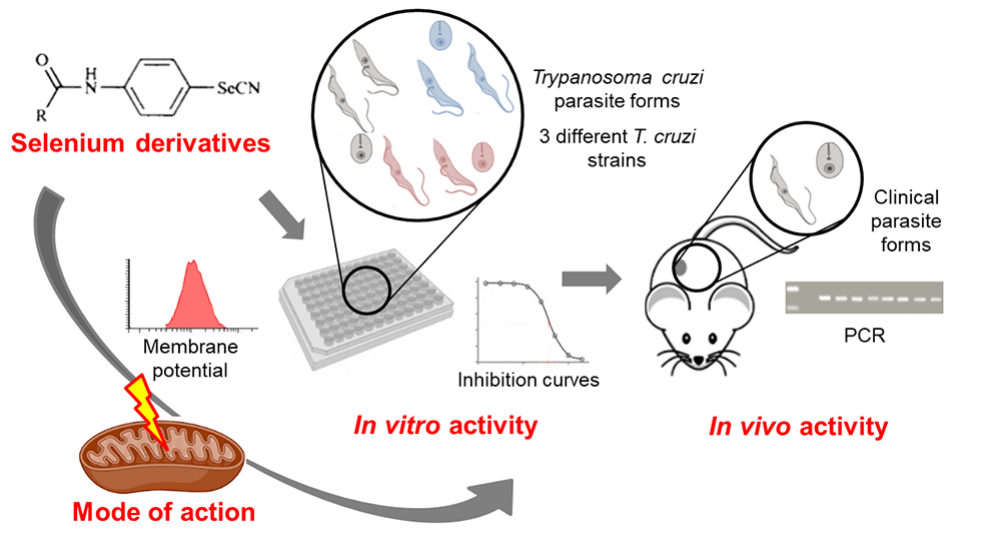

Chagas disease is a tropical infection caused by the protozoan parasite *Trypanosoma cruzi* and a global public health concern. It is a paradigmatic example of a chronic disease without an effective treatment. Current treatments targeting *T. cruzi* are limited to two obsolete nitroheterocyclic drugs, benznidazole and nifurtimox, which lead to serious drawbacks. Hence, new, more effective, safer, and affordable drugs are urgently needed. Selenium and their derivatives have emerged as an interesting strategy for the treatment of different prozotoan diseases, such as African trypanosomiasis, leishmaniasis, and malaria. In the case of Chagas disease, diverse selenium scaffolds have been reported with antichagasic activity *in vitro* and *in vivo*. On the basis of these premises, we describe the *in vitro* and *in vivo* trypanocidal activity of 41 selenocompounds against the three morphological forms of different *T. cruzi* strains. For the most active selenocompounds, their effect on the metabolic and mitochondrial levels and superoxide dismutase enzyme inhibition capacity were measured in order to determine the possible mechanism of action. Derivative **26**, with a selenocyanate motif, fulfills the most stringent *in vitro* requirements for potential antichagasic agents and exhibits a better profile than benznidazole *in vivo*. This finding provides a step forward for the development of a new antichagasic agent.

Chagas disease (CD) or American trypanosomiasis is a life-threatening tropical infection caused by the insect-transmitted protozoan parasite *Trypanosoma cruzi*. CD is an important public health problem in Latin America, being the major cause of morbimortality in many endemic regions: It affects 6–7 million people, causing about 14 thousand deaths annually, and it is hypothesized that about 100 million people are living at risk of infection worldwide.^1,2^ Blood-sucking triatomine bugs (vectors) are the main transmission route, although the oral route involving parasite-contaminated food and drink, the congenital route, as well as blood transfusion, transplantation, and laboratory accidents are also important.^3^ CD has recently spread to nonendemic areas as a result of migratory flows, particularly in the United States and Europe.^4,5^

In mammalian hosts, *T. cruzi* is an obligate intracellular parasite which can infect most nucleated cells.^6^ The parasites become widely disseminated in tissues and organs and can be detected in the bloodstream during the initial acute phase of CD. Following suppression by the adaptive immune response,^7^ CD progresses to a long-lasting asymptomatic chronic stage with an extremely low parasite burden. However, about 30% of patients will progress to a symptomatic chronic CD, developing cardiomyopathy and digestive tract megasyndromes, among others, for which there are few therapeutic options.^8^

Because of the gaps in knowledge about *T. cruzi*, the long-term nature of CD, and its complex pathology, no vaccines are available. Currently, the front-line chemotherapy used to treat CD is limited to two obsolete drugs for more than 50 years: benznidazole (BZN) and nifurtimox (NFX). These drugs lead to serious drawbacks, such as a range of toxic side-effects, extended treatment length and frequent treatment failures.^9,10^ Furthermore, the well-known cross-resistance–both drugs require metabolic activation within the parasite by the same mitochondrial nitroreductase^11^ – and the natural variation in susceptibility to drugs due to the extreme diversity of species^12^ make crucial the international effort aimed at developing new drugs against CD.

In this context, selenium (Se) and their derivatives have emerged as an interesting strategy for the treatment of different trypanosomiasis. Previous studies of our research group have shown that different selenocompounds exhibited promising activity against visceral leishmaniasis.^13−17^ Moreover, selenocompounds have displayed activity against malaria,^18^ African trypanosomiasis,^19^ or intestinal schistosomiasis.^20^ In the case of CD, several studies confirmed an association between the chronification of the disease and the decrease of Se plasma levels.^21^ In addition, Se supplementation therapies modulated the antioxidant, immune, and inflammatory responses, thus improving the intestinal megasyndrome,^22^ the placental immune response in pregnancy cases,^22^ and especially Chagasic cardiomyopathy problems.^23,24^ During the last years, diverse selenium scaffolds such as selenosemicarbazones,^25^ Se lapachones,^26^ Se quinones,^27^ Se naphtoquinones,^28^ and selenocyanates^29^ have been reported with antichagasic activity *in vitro* and *in vivo*. On the basis of these premises, and in connection with the interesting properties of selenium derivatives for the treatment of CD^30^ in the present contribution, we describe the *in vitro* and *in vivo* trypanocidal activity of 41 selenocompounds against the three morphological forms of three different *T. cruzi* strains. In order to expand the chemical space and the molecular diversity several selenoamides and selenophosphoramidates containing selenocyanate and diselenide motifs have been selected (Figure 1). For the most active and selective selenocompounds, its effect on the metabolite excretion, mitochondrial membrane potential, DNA or RNA alteration, and Fe-superoxide dismutase (SOD) enzyme inhibition capacity were measured in order to determine the possible mechanism of action.

**Figure 1:**
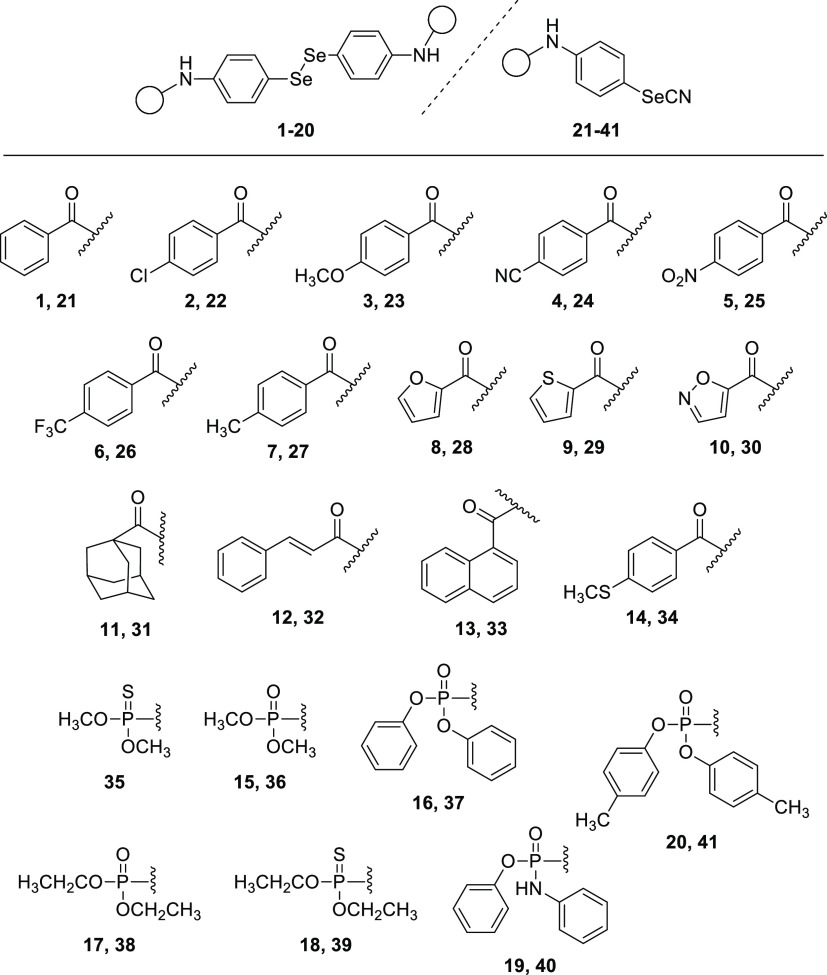
Structures of the 41 selenocompounds.

## Results and Discussion

### *In Vitro* Evaluation

Taking into account the parasite’s genetic diversity, drug resistance, the different sensitivities against moieties, and the CD target product profile established by the Drugs for Neglected Diseases *initiative* (DND*i*),^10,31^ compounds and BZN were evaluated against three different *T. cruzi* strains belonging to different discrete typing units (DTUs) associated with the human parasitosis. Moreover, the compounds’ effects on the viability and growth of the host cells was evaluated against Vero cells.

First, *in vitro* evaluation screening was performed using the extracellular epimastigotes because of their simple culture. The results, summarized in Table S1, are expressed as the inhibitory concentration 50 (IC_50_). Otherwise, selectivity index (SI) was calculated as the ratio between IC_50_ values of compounds against Vero cells relative to those against *T. cruzi* epimastigotes.

Compounds with SI values higher than 10 in at least one *T. cruzi* strain advanced to the *in vitro* activity assessment against the developed forms in vertebrate hosts (amastigotes and trypomastigotes); these are the relevant forms from a clinical point of view and the forms responsible for the acute and chronic CD.^32^ Therefore, compounds **25**, **26**, **28**, **29**, and **33** were selected to evaluate their IC_50_ and SI values against the amastigotes and trypomastigotes over the three strains (Tables 1 and 2). Interestingly, diselenide derivatives did not show activity at all against *T. cruzi* epimastigotes as it happens against *Leishmania infantum*.^33^ In contrast, 14 out of the 20 selenocyanate derivatives presented IC_50_ values lower than 20 μM at least in one of the strains, and 12 of them presented IC_50_ values lower than 10 μM in at least in one of the strains.

**Table 1:** Activity of Benznidazole and Selected Selenocompounds Tested against Cultured Amastigote and Trypomastigote forms of *Trypanosoma cruzi* Strains

	activity IC_50_ (μM)^a^					
	Arequipa strain		SN3 strain		Tulahuen strain	
comp.	am. forms	trypom. forms	am. forms	trypom. forms	am. forms	trypom. forms
BZN	8.3 ± 0.7	12.4 ± 1.1	16.6 ± 1.4	36.1 ± 3.1	10.0 ± 0.8	15.1 ± 1.3
**25**	49.3 ± 4.1	35.8 ± 5.8	nd	nd	>50.0	42.2 ± 3.9
**26**	5.4 ± 0.7	2.4 ± 0.3	3.9 ± 0.5	2.5 ± 0.3	4.9 ± 0.3	2.9 ± 0.2
**28**	3.2 ± 0.4	1.8 ± 0.1	1.7 ± 0.4	1.7 ± 0.2	2.7 ± 0.3	1.8 ± 0.2
**29**	15.4 ± 2.0	12.7 ± 1.2	nd	nd	nd	nd
**33**	nd	nd	16.9 ± 1.4	14.1 ± 1.1	nd	nd

a Inhibition concentration 50 (IC_50_): concentration (μM) required to inhibit 50% population, determined using GraphPad Prism 6. BZN, benznidazole. nd, not determined.

**Table 2:** Selectivity Index for Benznidazole and Selenocompounds on Amastigotes and Trypomastigotes of *Trypanosoma cruzi* Strains

	selectivity index (SI)^a^					
	Arequipa strain		SN3 strain		Tulahuen strain	
comp.	am. forms	trypom. forms	am. forms	trypom. forms	am. forms	trypom. forms
BZN	10	7	5	2	8	5
**25**	10 (1)	14 (2)	nd	nd	nd	nd
**26**	25 (3)	56 (8)	34 (7)	52 (26)	27 (3)	46 (9)
**28**	28 (3)	50 (7)	53 (11)	53 (26)	33 (4)	50 (10)
**29**	4 (0)	5 (1)	nd	nd	nd	nd
**33**	nd	nd	3 (1)	4 (2)	nd	nd

a Selectivity index (SI): IC_50_ Vero cells/IC_50_ developmental forms of the parasite. Data in parentheses refer to the number of times that compounds exceed the reference drug SI. BZN, benznidazole. nd, not determined.

The criteria established for this *in vitro* screening as the cutoff of this step were the same as those according to the literature:^34,35^ IC_50_ < 10 μM and SI > 50 (in at least two strains and one parasitic form). Therefore, compounds **26** and **28** were prioritized as potential compounds, and they were chosen for the subsequent *in vivo* evaluation. Moreover, these compounds were active against the three *T. cruzi* strains (including the BZN-resistant SN3 strain), devoid of drug resistance.

**Figure 2:**
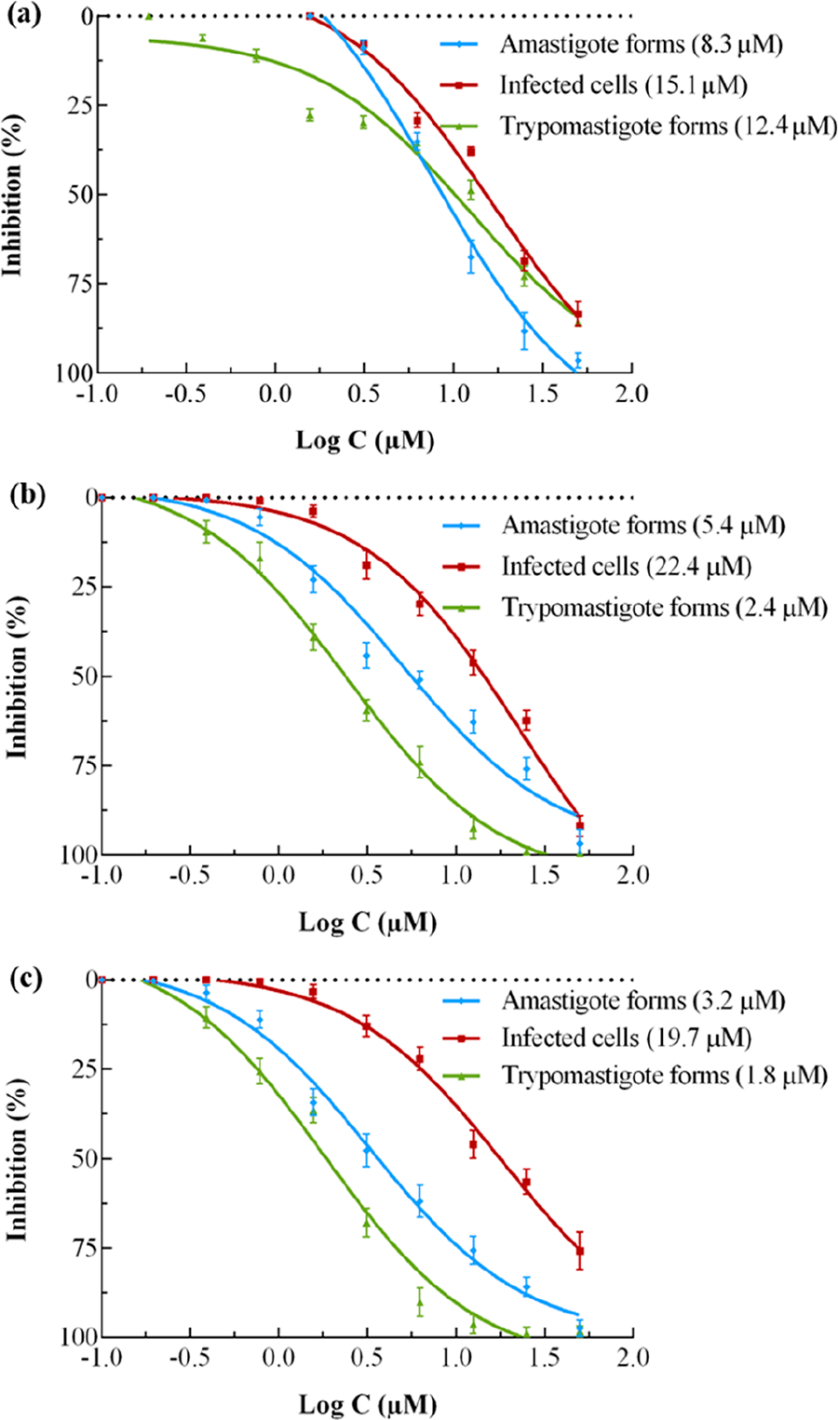
Infection of *Trypanosoma cruzi* Arequipa strain regarding the amastigotes and trypomastigotes and infected cells treated with (a) benznidazole, (b) **26**, and (c) **28**. Values constitute means of three independent experiments ± standard deviation. Data in parentheses refer to the IC_50_ value, calculated using GraphPad Prism 6.

Before going ahead with *in vivo* studies, the infection rates in Vero cells were evaluated by counting the infected cells after 72 h of treatment with compounds **26** and **28**. Results are summarized in Figure 2, together with the previously determined IC_50_ values against amastigotes and trypomastigotes of the Arequipa strains. It was found that the rates of infection (and the total number of amastigotes and trypomastigotes) decreased as the concentrations of compounds and BZN increased.

The average number of amastigotes per cell was also measured (Figure S1), giving an idea of the killing rate. This value was measured after 72 h of exposure at different concentrations of compounds and BZN, and the data show that compounds exhibit behavior similar to BZN, which is considered as a fast-acting compound.^36^ The number of amastigotes per cell gradually decreased in all cases after 72 h of exposure, highlighting that the effect of tested compounds at 50 μM reduced the number of amastigotes to practically zero. These data show that the compounds not only inhibit parasite multiplication but also cause its death. All these features are additional advantages since fast-acting and trypanocidal compounds can eliminate the parasite in a few doses,^37^ as well as act against the newfound quiescent or dormant amastigote form (responsible form of relapses after chemotherapy).^38^

### *In Vivo* Anti-*T. cruzi* Activity in BALB/c Mice

Considering the results obtained from *in vitro* assays, compounds **26** and **28** were selected as promising drugs for *in vivo* evaluation in BALB/c mice. These trials were performed using only the *T. cruzi* Arequipa strain in order to reduce the number of animals tests, as there were no significant differences for the other evaluated strains.

**Scheme 1:**
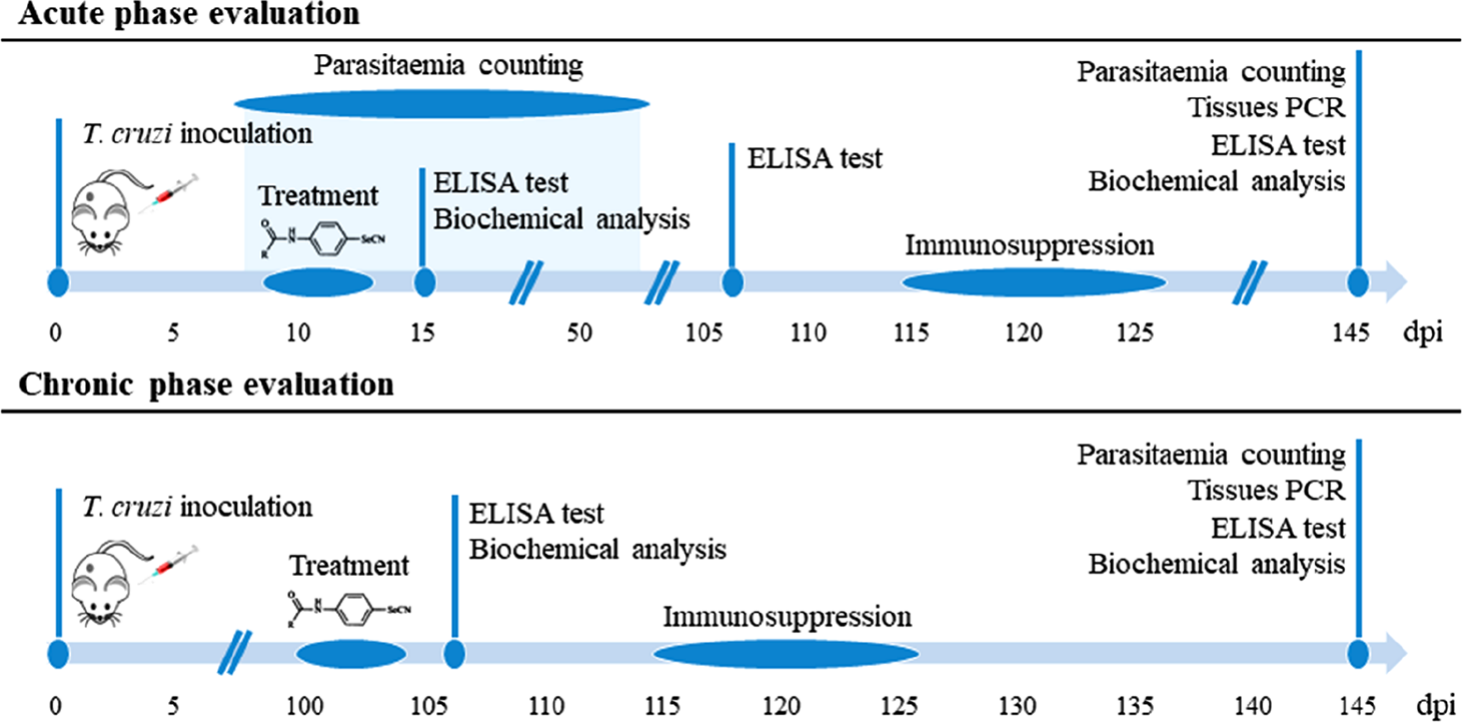
Timeline for All *In Vivo* Assays on BALB/c Mice for the Evaluation of Compounds in the Acute and Chronic Phases of Chagas Disease dpi = days post-infection.

As currently used clinical drugs presented variable activity in the acute and the chronic phase of the disease,^39^ compounds were evaluated in both phases according to the experiments established in Scheme 1. It should be noted that most *in vivo* testing has focused on acute CD, partially because it is simpler to monitor parasite burden.^40^ However, the ability to cure chronic CD is the main need from a clinical point of view.^31^ Therefore the different phase-specific drugs responses highlight that chronic CD should be the main research focus in animal models.

BALB/c mice were intraperitoneally infected according to a literature procedure.^41^ Compounds **26** and **28** or BZN were administered intraperitoneally from 9 days post-infection (dpi), once daily for 5 consecutive days at a dose of 20 mg·kg^1–^. This dosage represents the subcurative dose of BZN, so the experiment demonstrated whether the studies compound are more effective or not than the reference drug during the disease progress. In addition, it is shown that a compound showing a reasonable reduction in parasitaemia following 5 consecutive days of treatment can be considered a lead compound.^36^

**Figure 3:**
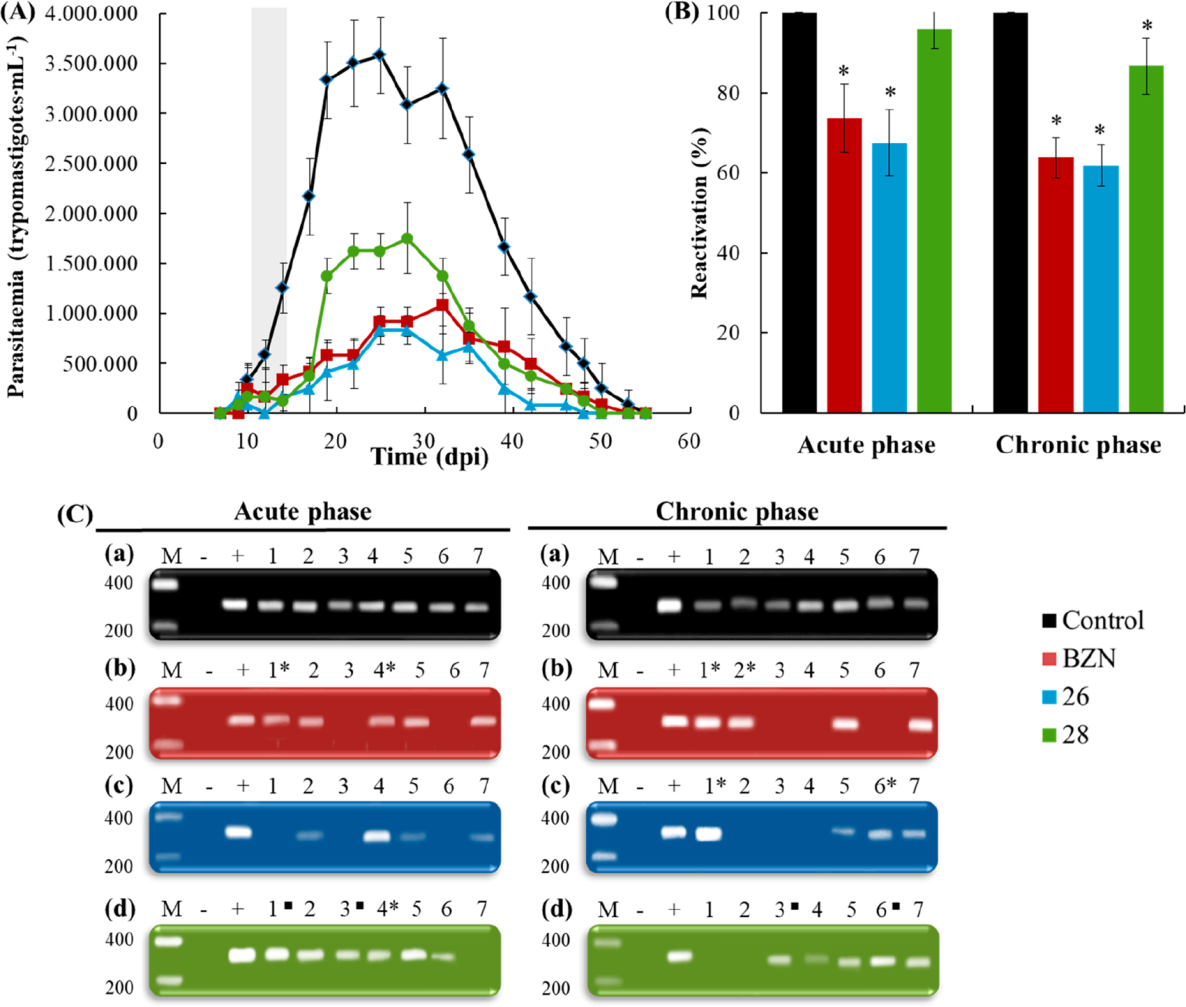
(A) Parasitaemia profiles of each group of mice infected with *Trypanosoma cruzi* and treated during the acute Chagas disease over a period of 60 days. Treatment days are represented in gray. Values are the means of three mice ± standard deviation. Significant differences between untreated and treated mice for α = 0.05. (B) Parasitaemia reactivation by fresh blood in the chronic Chagas disease after the immunosuppression cycles for each group of mice treated during the acute and chronic phases of the disease. Values are the means of three mice ± standard deviation. *, Significant differences between untreated and treated mice for α = 0.05. (C) PCR analysis of nine organs/tissues with the *Trypanosoma cruzi* spliced leader (SL) intergenic region sequence in the chronic Chagas disease for each group of mice treated during the acute and chronic phases of the disease. Lanes: (M) base pair marker, (−) PCR negative control, (+) PCR positive control, (1) adipose, (2) bone marrow, (3) brain, (4) esophagus, (5) heart, (6) lung, (7) muscle. ^■^, ^1^/_3_ of the corresponding organ/tissue PCR products showed banding on electrophoresis; *, ^2^/_3_ of the corresponding organ/tissue PCR products showed banding on electrophoresis.

First, parasitaemia levels of the four studies groups during the acute phase were determined by counting bloodstream trypomastigotes (BTs) as described in the “Methods” section (Figure 3A). Compound **28** showed a slight antiparasitic activity over the first month becoming similar to the BZN activity after 40 days. Compound **26** demonstrated a better activity profile compared to that of BZN, with low parasitaemia levels over the completely acute phase for 48 days. It is important to note that compound **26** caused an evident reduction of parasitaemia from the beginning of the treatment, even disappearing on 12 dpi. In addition, the parasitaemia of mice treated with compound **26** was resolved at 48 dpi, that is, 7 days before the parasitaemia of the untreated mice and those treated with BZN.

Second, the experimental cure was evaluated using a double checking widely used in animal models to evaluate the treatment effectiveness: immunosuppression (IS) and PCR of the target organs/tissues. Animals whose parasitaemia reactivation does not reappear and show negative PCR data after IS are considered cured.^42^

At 115 dpi, mice were immunosuppressed with cyclophosphamide monohydrate (CP) in order to assess the effectiveness of the treatments in acute and chronic CD. This procedure expands the residual infection to detectable levels in there is still presence of parasite after treatment.^43^Figure 3B shows the reactivation percentages of infection for each group of mice compared to those of the untreated (control) group after the immunosuppression. As observed, mice treated with BZN and compound **26** showed a similar parasitaemia reactivation. Compound **26** showed a reactivation of approximately 65 and 60% in acute and chronic phases respectively, being slightly more effective than BZN. However, mice treated with compound **28** showed a high parasitaemia reactivation (approximately 95 and 85% in acute and chronic phase, respectively). With this result, we hypothesized that compound **26** could be an effective antichagasic treatment. These results will be lately confirmed with the results obtained from tissue PCR.

In order to further evaluate the effectiveness of the compounds in acute and chronic CD, the presence of nested *T. cruzi* parasites in target organs/tissues^42^ was measured by PCR after necropsy (145 dpi). Figure 3C resumes the PCR results for the different target organs/tissues in the 4 mice groups in both phases. As expected, untreated mice (Figure 3C(a)) showed the presence of parasites in every analyzed organs/tissues for both phases. In mice treated with BZN, brain and lung appeared free of parasites for the acute phase, and brain, esophagus and lung appeared free of parasites for the chronic phase (28.6 and 42.9% of parasite-free organs/tissues, respectively). In accordance with previously obtained results, mice treated with compound **26** showed better results than those treated with BZN. In this case, adipose tissue, brain, and lung appeared free of parasites for the acute phase, and bone marrow, brain, esophagus for the chronic phase (42.9% of parasite-free organs/tissues in both phases). In contrast, treatment with compound **28** did not decrease enough parasite levels in the analyzed tissues. As observed, compound **26** showed the best trypanocidal activity after the double checking of the cure, even better than that shown by BZN, confirming the partial curative effect in both phases of CD at the tested dosage.

Otherwise, the immune response to *T. cruzi* infection was assessed by counting the immunoglobulin G (IgG) levels by indirect enzyme-linked immunosorbent assay (ELISA).^44^ The amount of IgG is directly associated with the parasitic load, and these experiments allowed the effectiveness of compounds evaluation in combination with the innate protection of the mice.^42^ The titer of anti-*T. cruzi* IgG in the different groups and the respective controls are shown in Figure S2. As observed, in the acute phase mice treated with compounds **26** or **28** showed lower IgG values than the treated with BZN at the beginning of the treatment becoming similar at the end, before and after IS. In the chronic phase, IgG values were similar between mice treated with compounds **26** and **28**, treated with BZN and the control mice. These results confirm the antichagasic activity of both compounds **26** and **28**, with compound **26** being more effective, especially in the acute phase. It has to be mentioned that the samples obtained after IS do not reflect data indicating infection rates, but rather confirm the IS suffered by the mice.

Moreover, splenomegaly is manifested in both acute and chronic phases on *T. cruzi* infected mice. This indicated the direct link between the enlargement of the spleen and the parasitic load. Figure S3 shows the weight percentage of the spleens for each group of mice. As observed, treated groups of mice demonstrated a smaller increase in the spleen weight when compared with the untreated (control) ones. Mice treated with compound **26** showed splenomegaly reduced by 49 and 38% in acute and chronic phases, respectively, and mice treated with compound **28** showed splenomegaly reduced by 37 and 48%, in comparison with the untreated (control) mice. Both compounds showed spleen weight percentage similar to the BZN-treated mice, with mice treated with compound **26** being more active in the acute phase, as previous results demonstrated.

Finally, the metabolic abnormalities associated with the treatment were determined by measuring kidney, heart, and liver biochemical markers (Table S2), including values for uninfected mice. Although most of the clinical parameters showed alteration at 2 days post-treatment, they returned to normal levels in the measurements obtained on the necropsy day. Moreover, it is noteworthy that none of the mice died or lost more than 10% body mass during and/or after treatment. The low toxicity allows these compounds to be studied at higher doses, establishing an improved treatment schedule based on pharmacokinetic studies in order to reach a sterile cure.

### Mode of Action (MoA) Studies

#### Metabolite Excretion

**Figure 4:**
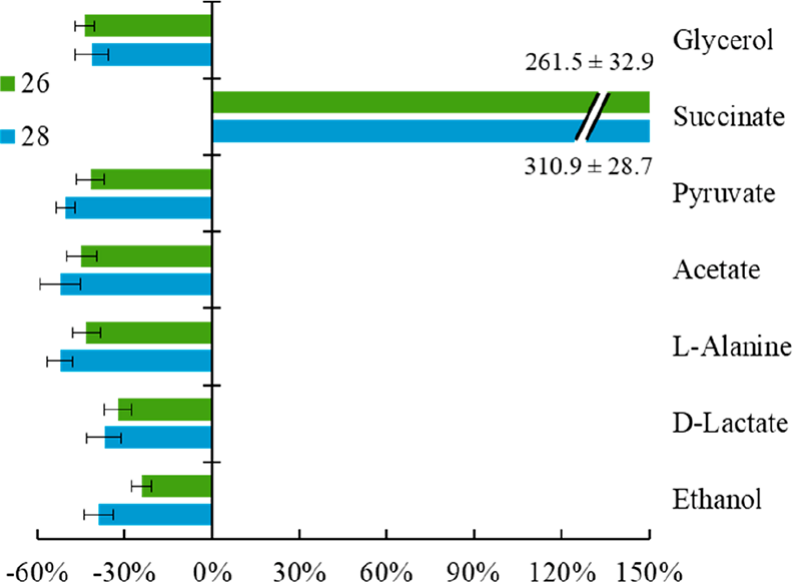
Variation among peaks of catabolites excreted by epimastigotes of *Trypanosoma cruzi* Arequipa strain exposed to **26** (green) and **28** (blue) at IC_25_ concentrations in comparison to control (untreated) parasites incubated 72 h. Values constitute means of three separate determinations ± standard deviation. Significant differences between untreated and treated parasites for α = 0.05.

*T. cruzi* parasites are known to significantly reduce glucose metabolism catabolites to pyruvate, acetate, and succinate, instead of degrading glucose to CO_2_ and water,^45^ so epimastigotes of *T. cruzi* Arequipa strain (untreated and treated with **26** and **28** at IC_25_ concentrations) were analyzed by ^1^H nuclear magnetic resonance (^1^H NMR) in order to measure different glucose metabolism catabolites. Figure 4 shows the percentage variation of excreted catabolites in treated parasites in comparison with control (untreated) parasites. As shown, excretion of all analyzed catabolites was altered, with succinate being the most altered for both treatments with values > 250%. According to the literature, an increase in excreted succinate may be closely related to a mitochondrial dysfunction.^45,46^ Therefore, mitochondrial stability assays were performed.

#### Mitochondrial Membrane Potential Measurement

**Figure 5:**
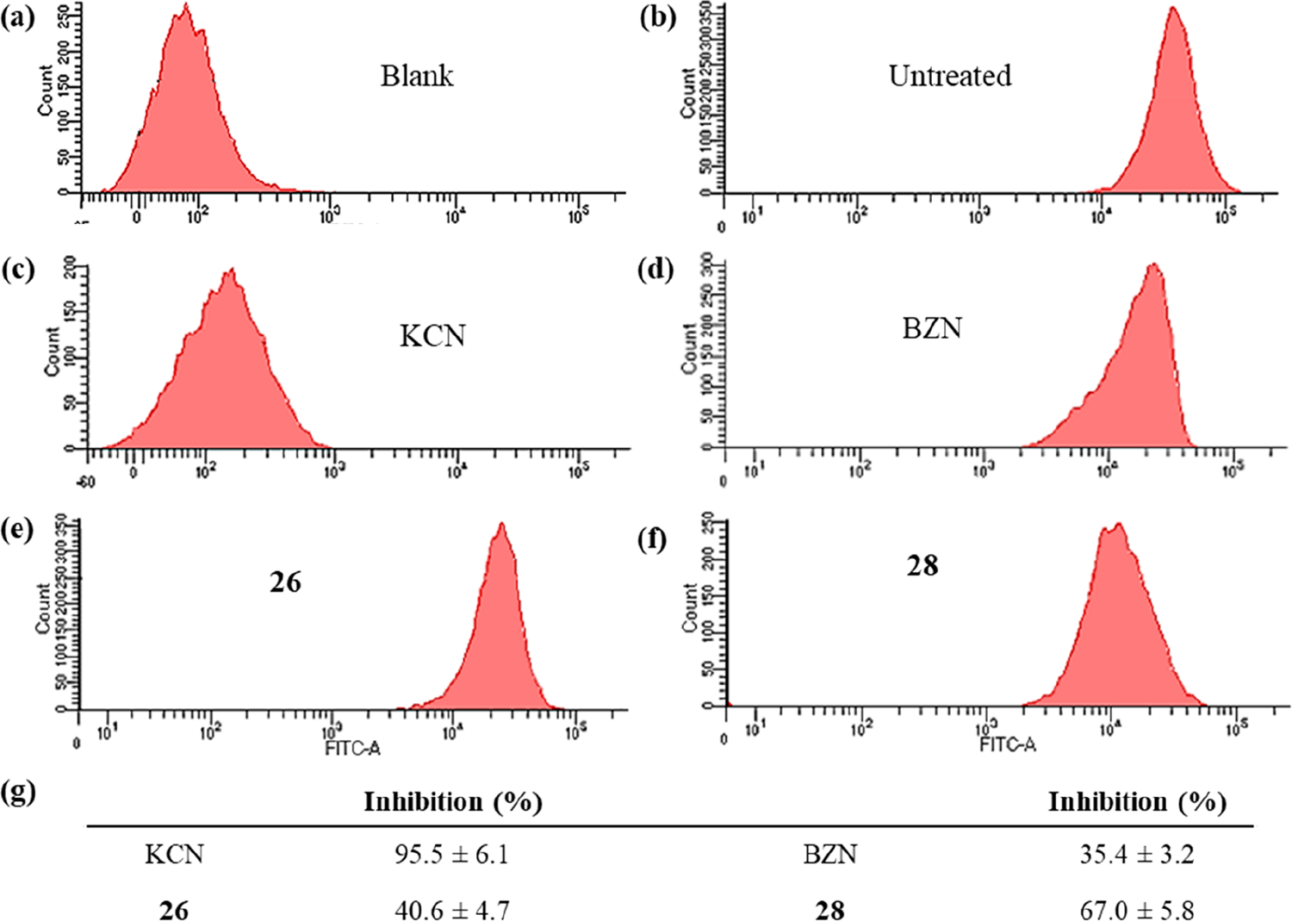
Mitochondrial membrane potential from epimastigotes of *Trypanosoma cruzi* Arequipa strain exposed for 72 h to benznidazole (BZN) and compounds at their IC_25_ concentrations: (a) blank, (b) untreated (control), (c) potassium cyanide (KCN), (d) BZN, (e) **26**, and (f) **28**. (g) Inhibition, in percentage, on mitochondrial membrane potential with respect to untreated parasites. Values constitute means of three separate determinations ± standard deviation. Significant differences between untreated and treated parasites for α = 0.05.

Mitochondria play an essential role in the maintenance of the electrochemical gradient and disturbances in the membrane potential could lead to less DNA replication, RNA transcription, and therefore cell apoptosis and/or necrosis. In view of the possible mitochondrial dysfunction, acridine orange (AO) and rhodamine 123 (Rho) staining were performed to evaluate the integrity of this organelle by flow cytometry. Figure 5 shows the measured percentage variation after treatment at IC_25_ concentrations, as stated before. BZN reduces the mitochondrial membrane potential (35.4%) due to its MoA.^47^ Parasites treated with compound **26** suffered a mitochondrial membrane depolarization of 40.6%, while parasites treated with compound **28** reached a higher depolarization of 67%. These results lead us to hypothesize that the antichagasic activity of the presented compounds may be related to an effect at the mitochondrial level: They could produce bioenergetic collapses, which precede *T. cruzi* death via necrosis in a mitochondrion-dependent manner, being the cause of the fast-acting trypanocidal activities of these compounds.

#### DNA and RNA Levels Measurement

As mentioned before, mitochondrial membrane potential alteration affects DNA replication and RNA transcription because of a decrease in ATP levels and a NADH/NAD^+^ imbalance.^45^ Thus, DNA and RNA levels were quantified by flow cytometry, with their percentages summarized in Figure S4. Significant alteration was observed in every treatment, with BZN, **26**, and **28** compared with the nontreated parasites. BZN inhibited nucleic acid levels up to 22%. Meanwhile, treatments with compounds **26** and **28** produced an inhibition of nucleic acid levels of 47 and 62%, respectively. These results bear out the possible MoA previously described. It must be noted that these inhibitions are due to not only an ATP deficit but also random nucleic acids degradation as a feature usually attributed to necrosis.^48^

#### *T. cruzi* Fe-SOD Enzyme Inhibition

**Figure 6:**
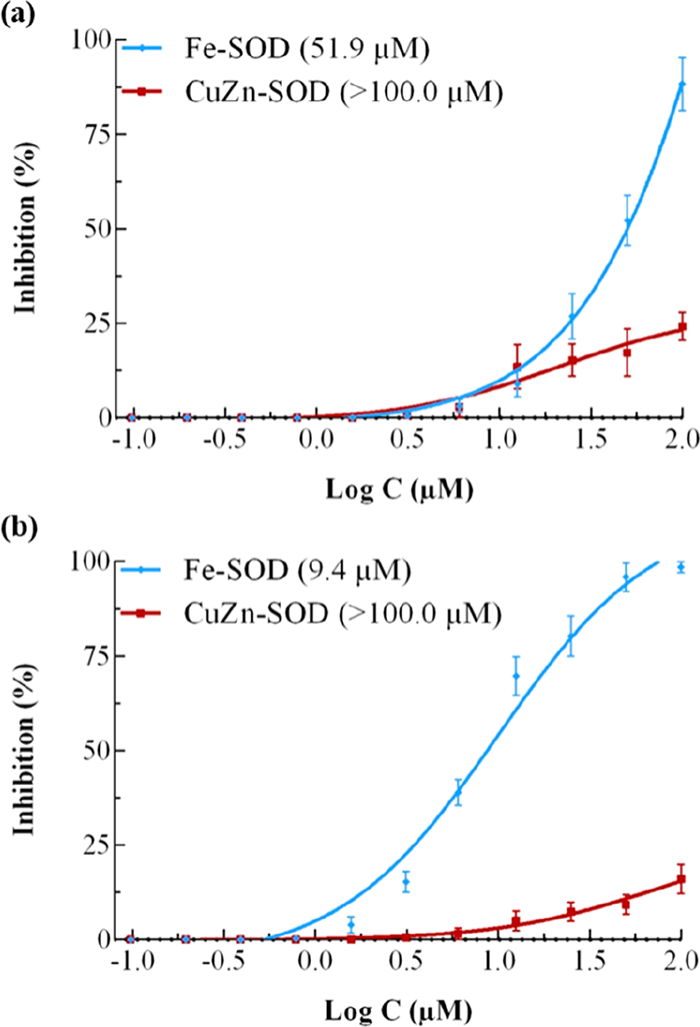
*In vitro* inhibition (%) of *Trypanosoma cruzi* Fe-SOD (activity 42.0 ± 3.8 U·mg^–1^) and human erythrocytes CuZn-SOD (activity 47.3 ± 4.1 U·mg^–1^) for (a) **26** and (b) **28**. Values constitute means of three separate determinations ± standard deviation. Data in parentheses refer to the IC_50_ value.

Previous studies of our research group demonstrated the selective inhibition against Fe-SOD of selenium derivatives,^13^ one of the most relevant targets for CD treatment. Therefore, in view of the previous results and the well-known antioxidant system modulator activity of these compounds,^33^ compounds **26** and **28** ability to inhibit Fe-SOD and/or Cu/SOD enzyme was assessed. Figure 6 reveals the inhibition curve of both compounds against Fe-SOD and human erythrocytes CuZn-SOD. Both compounds inhibit Fe-SOD enzyme activity, reaching IC_50_ values of 51.9 μM for compound **26** and 9.4 μM for compound **28**. Moreover, compounds did not reach 50% inhibition of Cu/SOD even at 100 μM. These results indicated the high selectivity of compound **28** against the parasitic enzyme, allowing us to propose it as one of the possible targets or MoA. Even when its trypanocidal effect can be ultimately ascribed to the inhibition of this enzyme, the possibility of multitarget activity should however not be rejected.

## Conclusion

In response to a dire need for new medications to treat CD, one chemical library of 41 leishmanicidal selenium amides and phophoramidate derivatives were screened against *T. cruzi*. The most active and selective compounds against the epimastigote form (compounds **25**, **26**, **28**, **29**, and **33**) were tested in the developed forms in vertebrate hosts (amastigotes and trypomastigotes). Taking into account the activity and selectivity of the derivatives, **26** and **28** were selected for the *in vivo* studies. Compound **26** exhibits a better profile than **28** and BZN, and it fulfils the most stringent *in vitro* requirements for potential antichagasic agents. It showed higher activity and lower toxicity than BZN after *in vivo* treatment, as indicated by different trials such as parasitemia monitoring, PCR, IS, or biochemical analysis. MoA analysis suggests a trypanocidal activity via necrosis in a mitochondrion-dependent manner through a bioenergetic collapse caused by a mitochondrial membrane depolarization. These findings suggest that these derivatives could be exploited and provide a step forward in the development of new antichagasic agents. It is worth considering higher doses and/or different treatment schedules, even combined therapies, to obtain a sterile cure.

## Methods

### Chemistry

The compound library consists of 41 compounds containing bis(4-aminophenyl)diselenide^33^ or 4-aminophenylselenocyanate^33^ entities as nucleus that have been decorated with aliphatic, cycloaliphatic, aromatic, and heteroaromatic substituents linked by an amide or phosphoramidate group. Structures are summarized in Figure 6. The synthesis and characterization of the molecules are reported in the literature.^33^ Briefly, amides derived from diselenide (compounds **4**, **11** and **13**) and selenocyanate (compounds **21**–**34**) were synthesized by an amide bond coupling with the corresponding amino groups of these scaffolds and the corresponding carbonyl of the acid chlorides.^33^ Compounds **1**–**3**, **5**–**10**, **12**, and **14** were prepared or by the reduction with sodium borohydride of the corresponding selenocyanate analogues.^33^ Finally, phosphoramidate derivatives were obtained by reaction between the phosphoryl chlorides and bis(4-aminophenyl)diselenide (compounds **15**–**20**) or 4-aminophenylselenocyanate (compounds **35**–**41**), respectively (unpublished). Briefly, derivatives **15**–**20** were obtained by reaction of a solution of bis(4-aminophenyl)diselenidein acetonitrile with the corresponding commercially available phosphoryl chlorides in a molar ratio (1:1) and triethylamine at 80 °C for 1 h under an inert atmosphere of nitrogen. Compounds **35**–**41** were obtained reacting 4-aminophenylselenocyanate, the corresponding phosphoryl chloride, in a molar ratio of 1:2, and trimethylamine in chloroform under nitrogen at 60 °C for 5 h. Phosphoramidate derivatives (**15**–**20** and **35**–**41**) were purified by flash chromatography using different gradients of hexane/ethyl acetate as eluents.

### *In Vitro* Trypanocidal Activity

#### Screening against Extracellular Epimastigotes

*T. cruzi* epimastigotes of three different strains [Arequipa strain (MHOM/Pe/2011/Arequipa, DTU V),^42^ SN3 strain (IRHOD/CO/2008/SN3, DTU I)^49^ and Tulahuen strain (TINF/CH/1956/Tulahuen, DTU VI)]^42^ were cultured at 28 °C in Gibco RPMI 1640 medium supplemented with 10% (*v*/*v*) heat-inactivated fetal bovine serum (FBS), 0.03 M hemin, and 0.5% (*w*/*v*) BBL trypticase.^50^

Trypanocidal activity against epimastigotes was tested as previously described.^42^ In short, 5 × 10^5^ epimastigotes·mL^–1^ were treated by adding the tested selenocompounds and BZN to the corresponding well at a concentration range of 50–0.5 μM in 96-well plates (200 μL·well^–1^) for 48 h. Untreated controls were also included. Subsequently, resazurin sodium salt (Sigma-Aldrich) was added, and the cells were incubated for further 24 h. Finally, the absorbance was measured using a Sunrise absorbance reader, and the trypanocidal activity was expressed as the IC_50_ using GraphPad Prism 6 software. Each compound concentration was tested in triplicate in three independent experiments.

#### Cytotoxicity Test

Mammalian Vero cells (EACC No. 84113001), cultured as previously reported,^41^ were used to determine the cytotoxicity of the selenocompounds.^42^ In short, 1.25 × 10^4^ Vero cells·mL^–1^ were treated by adding the tested selenocompounds and BZN at a concentration range of 2000–50 μM in 96-well plates (200 μL·well^–1^) at 37 °C for 48 h. Untreated controls were also included. Subsequently, resazurin sodium salt (Sigma-Aldrich) was added, and the cells were incubated for a further 24 h. Finally, cell viability was determined following the same procedure as described to assess the trypanocidal activity in the epimastigotes. Each compound concentration was tested in triplicate in three independent experiments.

#### Screening against Intracellular Amastigotes and Infected Cells

Trypanocidal activity against amastigotes was determined according to the literature reported previously.^41^ In short, 1 × 10^4^ Vero cells·well^–1^ were seeded in 24-well plates and then infected with culture-derived trypomastigotes (obtained as previously described)^42^ at a multiplicity of infection (MOI) ratio of 1:10. After 24 h of infection, nonphagocyted trypomastigotes were washed, and the plates were treated by adding the tested selenocompounds and BZN to the corresponding well at a concentration range of 50–0.1 μM in 500 μL·well^–1^. Untreated controls were also included. After 72 h of incubation, the trypanocidal effect was determined based on the counting of amastigotes and infected cells in methanol-fixed and Giemsa-stained preparations, and the activity was expressed as the IC_50_ using GraphPad Prism 6 software. Each compound concentration was tested in triplicate in three independent experiments.

#### Screening against Bloodstream Trypomastigotes

BTs (2 ×10^6^ BTs·mL^–1^, obtained as previously described from infected BALB/c mice)^51^ were treated by adding the tested selenocompounds and BZN at a concentration range of 50–0.1 μM in 96-well plates (200 μL·well^–1^) at 37 °C. Untreated controls were also included. After 24 h of treatment, resazurin sodium salt (Sigma-Aldrich) was added, and the cells were incubated for a further 4 h. Finally, trypanocidal activity was determined following the same procedure as described to assess the trypanocidal activity in the epimastigotes. Each compound concentration was tested in triplicate in three independent experiments.

### *In Vivo* Trypanocidal Activity on BALB/c Mice

#### Ethics Statement

All animal work and maintenance was performed under RD53/2013 and approved by the Ethics Committee on Animal Experimentation (CEEA) of the University of Granada, Spain.

#### Infection and Treatment

Female BALB/c mice aged 10–12 weeks and with a size of 20–24 g were divided into five groups (*n* = 3 per group): 0, negative control group (uninfected and untreated mice); I, positive control group (infected and untreated mice); II, BZN group (infected mice treated with BZN); III, **26** group (infected mice treated with **26**); and IV, **28** group (infected mice treated with **28**).

Mice were infected by intraperitoneal injection of 5 × 10^5^ BTs of *T. cruzi* Arequipa strain per mouse in 200 μL PBS.^42^ Subsequently, the treatment was intraperitoneally administered (∼200 μL) once daily for 5 consecutive days.

The tested selenocompounds and BZN were prepared at 2 mg·mL^–1^ in an aqueous suspension vehicle containing 5% (*v*/*v*) DMSO and 0.5% (*w*/*v*) hydroxypropyl methylcellulose, as previously reported.^52^ Compounds (20 mg·kg^–1^ per day) were administered for 5 consecutive days (total dosages of 100 mg·kg^–1^), and vehicle only was administered in the negative and positive control groups. Treatments began when the infection was confirmed (9 dpi) for acute-phase-treated mice, and when it was established that the animals moved into the chronic phase (100 dpi, for chronic-phase-treated mice; Scheme 1).

#### Monitoring of Parasitaemia during the Acute Phase Treatment

Parasitemia levels were measured by counting BTs from peripheral blood drawn from the mandibular vein and diluted at a ratio of 1:100, as previously described.^42^ Fresh blood microscopic examination was performed until the day the parasitemia was undetected (Scheme 1). Parasitemia was expressed as parasites·mL^–1^.

#### Immunosuppression

Immunosuppression was performed by intraperitoneal injection of three doses of 200 mg·kg^–1^ of ISOPAC CP at 3–4 day intervals (Scheme 1), as previously reported.^53^ Mice were closely monitored for side effects or for secondary infections due to immunosuppression. Within 7 days after the last CP injection, parasitemia reactivation was determined by counting BTs according the procedure described for parasitemia in the acute phase.^42^

#### Mice Sacrifice, Blood Collection, and Organs/Tissues Extraction

On 145 dpi, the mice were euthanized using CO_2_, followed by exsanguination via cardiac puncture, and blood was collected. Nine target organs/tissues (adipose, bone marrow, brain, esophagus, heart, lung, muscle, spleen, and stomach) were then harvested^42^ and perfused with prewarmed PBS to avoid contamination with BTs.^54^ Finally, they were stored at −80 °C until DNA extraction. In addition, spleens were weighed to assess splenomegaly.^42^

#### Tissue DNA Extraction, PCR, and Electrophoresis

DNA extraction of the post-mortem organs/tissues was performed using Wizard Genomic DNA Purification Kit,^41^ and the extracted DNA was subjected to amplification by PCR based on the spliced leader (SL) intergenic region sequence of *T. cruzi* (for detailed description, see literature reported previously).^55^ Finally, the PCR products were resolved by electrophoresis on a 2% agarose gel (containing GelRed nucleic gel stain) for 90 min at 90 V.

#### ELISA Test

Serum samples were obtained from blood collected on several days post-infection (Scheme 1), processed according the method previously described,^42^ and aliquoted to the ELISA test and biochemical analysis, as mentioned below.

Circulating antibodies in serum against both antigens were quantitatively evaluated in triplicate by the ELISA test in 96-well plates using diluted serum samples (1:80 in PBS), as previously described.^41^

#### Toxicity Test by Clinical Analysis

Serum samples obtained from blood in several days post-infection (Scheme 1) were sent to the Biochemical Service (University of Granada) to measure a series of biochemical parameters with the commercial Cromakit using a clinical chemistry analyzer (BS-200, Shenzhen Mindray Biomedical Electronics Co., LTD), as previously described.^41^

### Mode of Action Studies

#### ^1^H NMR Analysis of Excreted Metabolites

*T. cruzi* Arequipa (5 ×10^5^ epimastigotes·mL^–1^) were treated by adding the tested compounds at IC_25_ concentrations in 25 cm^2^ cell culture flasks at 28 °C for 72 h. Untreated controls were also included. Cultures were then centrifuged and filtered, and the metabolites of the supernatants were analyzed using a ^1^H NMR spectrometer (Varian Direct Drive 500 MHz Bruker) with AutoX probe, D_2_O as solvent, and 2,2-dimethyl-2-silapentane-5-sulfonate as the reference signal.^56^ Chemical shifts were expressed in parts per million (ppm), and analyses were conducted as previously reported.^57^

#### Flow Cytometry Analysis of Mitochondrial Membrane Potential and Nucleic Acid Levels

The untreated and treated epimastigotes of *T. cruzi* Arequipa described in the ^1^H NMR analysis were collected by centrifugation, washed three times in PBS, and stained with 10 mg·mL^–1^ Rho (Sigma-Aldrich) or AO (Sigma-Aldrich) dyes in 0.5 mL of PBS for 20 min.^58^ Control epimastigotes with a fully depolarized mitochondrion were obtained by incubation for 40 min with 10 Mm KCN prior to Rho loading.^59^ Nonstained parasites were also included. After the elapsed time, epimastigotes were processed and analyzed by flow cytometry as previously reported.^57^

#### SOD Enzymatic Inhibition Analysis

The *in vitro* activities of either excreted Fe-SOD from *T. cruzi* (obtained as previously described)^57^ and commercial copper/zinc superoxide dismutase (Cu/Zn-SOD) from human erythrocytes (Sigma-Aldrich) were evaluated using the method previously described^60^ after incubation with a concentration range of compound from 100 to 0.1 μM.

#### Statistical Analyses

Statistical analyses were performed with the SPSS v21.0 software (IBM Corp; Armonk, NY). The *t*-test for paired samples was used to verify whether there were differences between the assays used. Differences were considered significant when the *p*-value was less than 0.05. Also statistical studies based on contingency tables (prevalence) were conducted.

## Supporting Information Available

Number of amastigotes of *T. cruzi* Arequipa strain per Vero cell. Anti-*T. cruzi* immunoglobulin G levels at different days postinfection. Weight percentage of spleens in the chronic Chagas disease. Nucleic acids levels of *T. cruzi* Arequipa strain. Activity of benznidazole and selenocompounds tested against cultured epimastigote form of *T. cruzi* strains, and toxicity against cultured Vero cells. Clinical analysis. (PDF)

## Abbreviations

AO: acridine orange
BTs: bloodstream trypomastigotes
BZN: benznidazole
CD: Chagas disease
CP: cyclophosphamide monohydrate
DND*i*: Drug for Neglected Diseases *initiative*
DTU: discrete typing unit
ELISA: enzyme-linked immunosorbent assay
FBS: fetal bovine serum
IC_50_: inhibitory concentration 50
Ig: immunoglobulin
MoA: mode of action
MOI: multiplicity of infection
NFX: nifurtimox
Rho: rhodamine 123
Se: selenium
SI: selectivity index
SL: splice leader
SOD: superoxide dismutase
^1^H NMR: ^1^H nuclear magnetic resonance
