# Supplementary material for: Selenium Derivatives as Promising Therapy for Chagas Disease: In Vitro and In Vivo Studies
Source: ACS Infect Dis. 2021 Apr 19;7(6):1727–38. doi: 10.1021/acsinfecdis.1c00048 (PMC8480776; doi:10.1021/acsinfecdis.1c00048)
Supplement: Supplementary file 1 — id1c00048_si_001.pdf [file id1c00048_si_001.pdf]

SUPPORTING INFORMATION

Selenium derivatives as promising therapy for Chagas disease: *In vitro* and *in vivo* studies

Rubén Martín-Escolano[ϒ], Mikel Etxebeste-Mitxeltorena[‡,§,¥], Javier Martín-Escolano[†], Daniel Plano[‡,§,¥], María J. Rosales[†], Socorro Espuelas[‡,§,¥], Esther Moreno[‡,§,¥], Manuel Sánchez Moreno[†], Carmen Sanmartín[‡,§,¥], Clotilde Marín[†,*]

[ϒ]Laboratory of Molecular & Evolutionary Parasitology, RAPID group, School of Biosciences, University of Kent, Canterbury, CT2 7NJ, UK.

[†]Department of Parasitology, Instituto de Investigación Biosanitaria (ibs.Granada), Hospitales Universitarios De Granada/University of Granada, Severo Ochoa s/n, 18071 Granada, Spain.

[‡]Universidad de Navarra, Facultad de Farmacia y Nutrición, Departamento de Tecnología y Química Farmacéuticas, Irunlarrea, 1. E-31008 Pamplona, Spain

[§]Instituto de Salud Tropical, Universidad de Navarra, ISTUN, Irunlarrea, 1. E-31008 Pamplona, Spain

[¥]Instituto de Investigaciones Sanitarias de Navarra (IdiSNA), Irunlarrea, 1. E-31008 Pamplona, Spain

Corresponding Author

*Clotilde Marín

E-mail: cmaris@ugr.es

Supporting Information

Figure S1. Number of amastigotes of *Trypanosoma cruzi* Arequipa strain per Vero cell.

Figure S2. Anti-*Trypanosoma cruzi* immunoglobulin G levels at different days post-infection (dpi).

Figure S3. Weight percentage of spleens in the chronic Chagas disease.

Figure S4. Nucleic acids levels of *Trypanosoma cruzi* Arequipa strain.

Table S1. Activity of benznidazole and selenocompounds tested against cultured epimastigote form of *Trypanosoma cruzi* strains, and toxicity against cultured Vero cells.

Table S2. Clinical analysis.

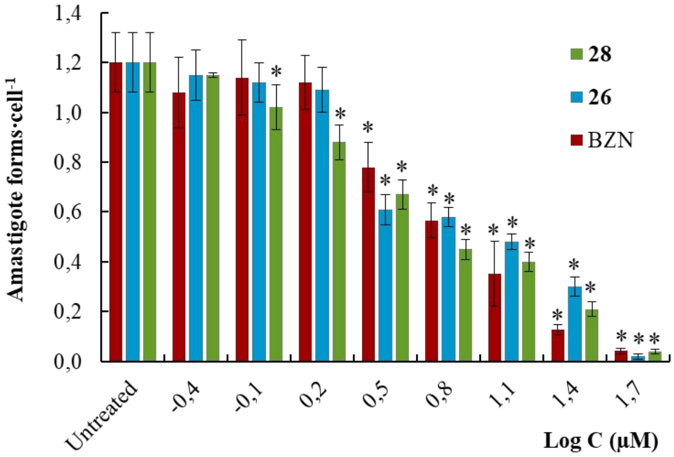

Figure S1. Number of amastigotes of *Trypanosoma cruzi* Arequipa strain per Vero cell exposed to benznidazole (BZN), **26** and **28**. Values constitute means of three independent experiments ± standard deviation. * Significant differences between untreated and treated parasites for α = 0.05.

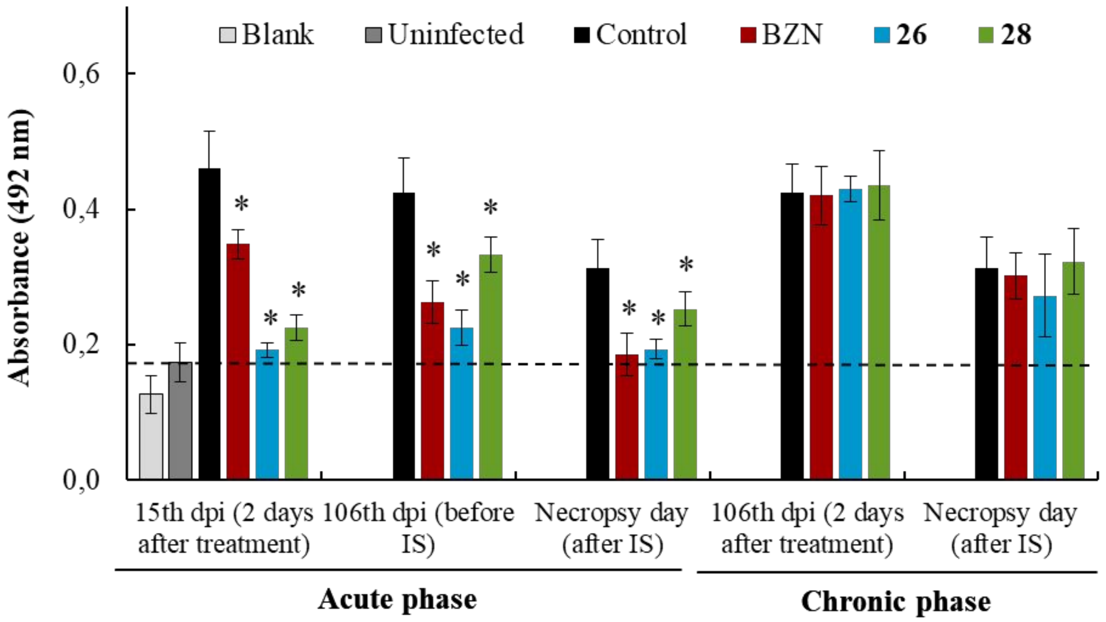

Figure S2. Anti-*Trypanosoma cruzi* immunoglobulin G levels, expressed in absorbance at 492 nm, at different days post-infection (dpi) for each group of mice treated during the acute and chronic phases of Chagas disease: control (untreated), benznidazole (BZN), **26** and **28**. Blank and uninfected mice are also included. Dashed line shows the cut off for uninfected mice. Values are the means of three mice ± standard deviation. (IS) immunosuppression. * Significant differences between untreated and treated mice for $\alpha = 0.05$.

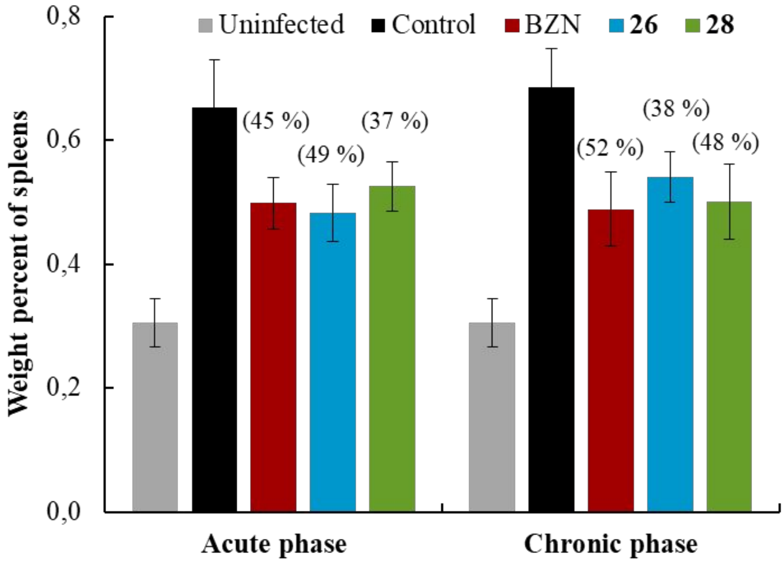

Figure S3. Weight percentage of spleens in the chronic Chagas disease for each group of mice treated during the acute and chronic phases of the disease: control (untreated), benznidazole (BZN), **26** and **28**. Values are the means of three mice ± standard deviation. Data in brackets refer to the reduction of splenomegaly in comparison to the control. Significant differences between untreated and treated mice for $\alpha = 0.05$.

(a)

(b)

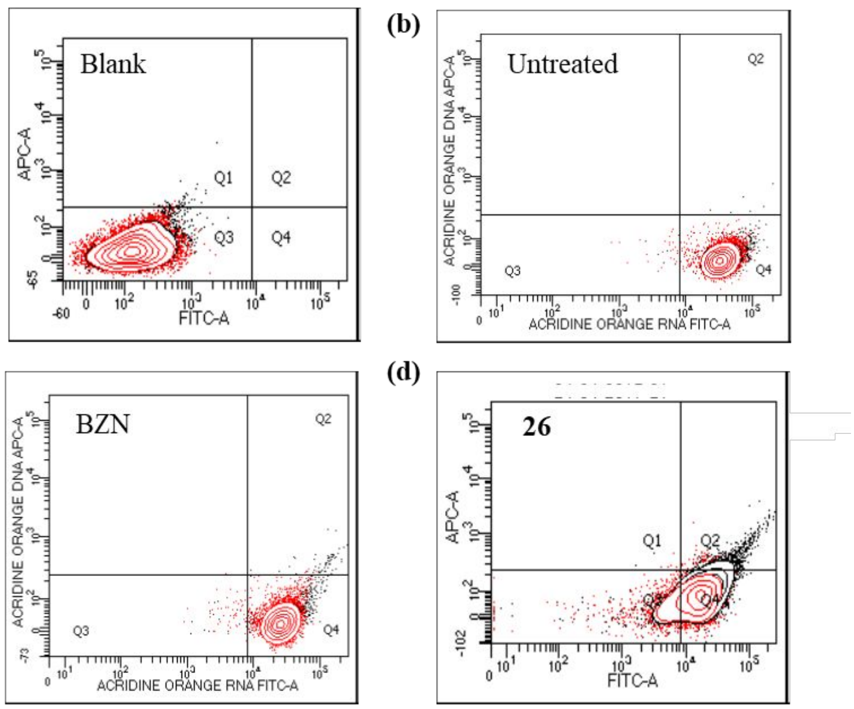

(e)

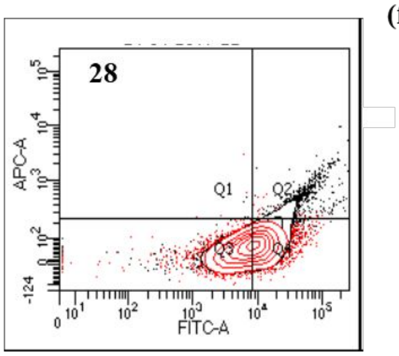

(f)

	Inhibition (%)
BZN	22.4 ± 3.6
**26**	47.6 ± 5.3
**28**	62.5 ± 6.4

Figure S4. Nucleic acids levels of *Trypanosoma cruzi* Arequipa strain exposed to benznidazole (BZN) and compounds at their IC_{25} concentrations incubated 72 h: (a) blank, (b) untreated (control), (c) BZN, (d) **26**, (e) **28**. (f) Inhibition, in percentage, in the nucleic acids levels with respect to untreated parasites. Values constitute means of three separate determinations ± standard deviation. Significant differences between untreated and treated parasites for $\alpha = 0.05$.

Table S1. Activity of benznidazole and selenocompounds tested against cultured epimastigote form of *Trypanosoma cruzi* strains, and toxicity against cultured Vero cells.

Compound	Activity IC50 (μM) [a]			Toxicity IC_{50} (μM) [b] Vero cell	Selectivity index (SI) [c]		
	Arequipa strain	SN3 strain	Tulahuen strain		Arequipa strain	SN3 strain	Tulahuen strain
**BZN**	16.9 ± 1.8	36.2 ± 2.4	19.7 ± 1.7	80.4 ± 7.1	5	2	4
**1**	>50.0	>50.0	>50.0	800.2 ± 44.0	nd	nd	nd
**2**	>50.0	>50.0	>50.0	622.1 ± 41.9	nd	nd	nd
**3**	>50.0	>50.0	>50.0	252.4 ± 21.6	nd	nd	nd
**4**	>50.0	>50.0	>50.0	280.0 ± 34.2	nd	nd	nd
**5**	>50.0	>50.0	>50.0	552.4 ± 43.1	nd	nd	nd
**6**	>50.0	>50.0	>50.0	535.8 ± 39.2	nd	nd	nd
**7**	>50.0	>50.0	>50.0	341.1 ± 28.3	nd	nd	nd
**8**	>50.0	>50.0	>50.0	1248.3 ± 97.7	nd	nd	nd
**9**	>50.0	48.0 ± 6.4	>50.0	<50.0	nd	nd	nd
**10**	>50.0	>50.0	>50.0	212.4 ± 18.7	nd	nd	nd
**11**	>50.0	>50.0	>50.0	225.6 ± 19.1	nd	nd	nd
**12**	>50.0	>50.0	>50.0	280.2 ± 24.4	nd	nd	nd
**13**	>50.0	>50.0	>50.0	98.2 ± 12.8	nd	nd	nd
**14**	>50.0	>50.0	>50.0	407.2 ± 17.8	nd	nd	nd
**15**	19.6 ± 2.0	12.5 ± 1.1	14.4 ± 1.7	<50.0	nd	nd	nd
**16**	>50.0	>50.0	>50.0	147.1 ± 11.4	nd	nd	nd
**17**	>50.0	>50.0	>50.0	182.6 ± 20.0	nd	nd	nd
**18**	>50.0	>50.0	>50.0	181.5 ± 14.3	nd	nd	nd
**19**	>50.0	>50.0	>50.0	<50.0	nd	nd	nd
**20**	>50.0	>50.0	>50.0	<50.0	nd	nd	nd
**21**	>50.0	37.1 ± 4.6	>50.0	<50.0	nd	nd	nd
**22**	36.2 ± 2.8	5.4 ± 0.7	19.4 ± 1.4	<50.0	nd	nd	nd
**23**	34.5 ± 2.7	4.1 ± 0.4	17.6 ± 2.5	<50.0	nd	nd	nd
**24**	30.5 ± 1.9	9.8 ± 1.2	15.4 ± 2.1	70.8 ± 5.4	2 (0)	7 (3)	5 (1)
**25**	26.3 ± 2.1	>50.0	25.3 ± 4.1	516.3 ± 42.2	20 (4)	nd	20 (5)
**26**	1.6 ± 0.3	2.1 ± 0.3	2.6 ± 0.4	133.9 ± 11.3	84 (17)	64 (32)	52 (13)
**27**	4.5 ± 0.7	4.2 ± 0.3	4.9 ± 0.3	<50.0	nd	nd	nd

**28**	4.8 ± 0.4	3.8 ± 0.4	4.6 ± 0.3	89.3 ± 7.5	19 (4)	24 (12)	19 (5)
**29**	5.4 ± 0.8	8.9 ± 1.1	6.6 ± 0.4	60.9 ± 8.7	11 (2)	7 (3)	9 (2)
**30**	9.8 ± 1.0	24.1 ± 2.3	15.3 ± 1.1	86.0 ± 6.7	9 (2)	4 (2)	6 (2)
**31**	4.6 ± 0.5	12.5 ± 1.6	8.1 ± 0.9	<50.0	nd	nd	nd
**32**	7.4 ± 0.9	4.8 ± 0.8	7.9 ± 0.5	<50.0	nd	nd	nd
**33**	9.4 ± 1.2	4.8 ± 0.6	10.8 ± 1.2	52.7 ± 4.9	6 (1)	11 (5)	5 (1)
**34**	16.4 ± 2.0	21.0 ± 2.7	29.1 ± 3.1	72.6 ± 9.9	4 (1)	3 (2)	2 (1)
**35**	38.2 ± 3.5	30.5 ± 2.8	41.5 ± 3.8	<50.0	nd	nd	nd
**36**	28.1 ± 3.2	4.3 ± 0.7	18.4 ± 1.4	<50.0	nd	nd	nd
**37**	20.1 ± 2.8	30.3 ± 3.2	22.3 ± 3.0	<50.0	nd	nd	nd
**38**	>50.0	>50.0	>50.0	<50.0	nd	nd	nd
**39**	38.9 ± 5.5	>50.0	>50.0	<50.0	nd	nd	nd
**40**	41.1 ± 4.4	>50.0	>50.0	<50.0	nd	nd	nd
**41**	>50.0	43.2 ± 5.3	29.9 ± 2.1	<50.0	nd	nd	nd

[a] Inhibition concentration 50 (IC_{50}), concentration (μM) required to inhibit 50% growth, determined using GraphPad Prism 6. [b] Towards Vero cells. [c] Selectivity index (SI), IC_{50} Vero cells / IC_{50} developmental forms of the parasite. Data in brackets refer to the number of times that compounds exceed the reference drug SI. Values are the means of three independent experiments ± standard deviation. BZN, benznidazole. nd, not determined.

Table S2. Clinical analysis determined at different days post-infection in groups of mice infected with *Trypanosoma cruzi* and treated with benznidazole and compounds.

		**Kidney marker profile**		**Heart marker profile**		**Liver marker profile**			
		**Urea (mg/dL)**	**Uric acid (mg/dL)**	**CK-MB (U/L)**	**LDH (U/L)**	**AST/GOT (U/L)**	**ALT/GPT (U/L)**	**Total bilirubin (mg/dL)**	**ALP (U/L)**
**Uninfected mice (n = 6)**		38 [32–40]	4.4 [4.0 –5.1]	492 [150–630]	3121 [2505–3851]	153 [132–177]	50 [46–62]	0.23 [0.22 –0.31]	165 [141–192]
**Treatment in acute phase**	15th dpi (Control) (n = 3)	31	4.3	535	3275	167	60	0.23	180
	15th dpi and BZN (2 days after treatment) (n = 3)	--	----	=	=	++++	++	++++	=
	15th dpi and **26** (2 days after treatment) (n = 3)	--	----	++++	+	++	++	++	+
	15th dpi and **28** (2 days after treatment) (n = 3)	--	----	++++	-	++	++	++	+
	Necropsy day of mice (Control) (n = 3)	34	4.0	496	2761	179	49	0.21	161
	Necropsy day of mice and BZN (n = 3)	--	-	-	=	+	=	++	=
	Necropsy day of mice and **26** (n = 3)	=	=	=	=	=	++	+	++
	Necropsy day of mice and **28** (n = 3)	=	-	=	=	+	++	=	+
**Treatment in chronic phase**	106th dpi (Control) (n = 3)	45	5.7	751	5951	260	57	0.25	167
	106th dpi and BZN (2 days after	-	-	--	--	+	++	+	++++

	treatment) (n = 3)								
	106th dpi and **26** (2 days after treatment) (n = 3)	---	-	+	=	+++	++	+++	=
	106th dpi and **28** (2 days after treatment) (n = 3)	=	=	++	=	=	+	=	+
	Necropsy day of mice (Control) (n = 3)	37	4.8	538	6679	286	64	0.20	149
	Necropsy day of mice and BZN (n = 3)	=	-	--	--	+	++	++	=
	Necropsy day of mice and **26** (n = 3)	=	-	+	=	=	=	++	=
	Necropsy day of mice and **28** (n = 3)	=	=	+	=	+	=	=	=

CK-MB, creatine kinase-muscle/brain. LDH, lactate dehydrogenase. AST/GOT, aspartate aminotransferase. ALT/GPT, alanine aminotransferase. ALP, Alkaline phosphatase. BZN, benznidazole. dpi, day post-infection.

Key: =, variation ≤ 10%; +/-, 10-20% increase/decrease over the range; ++/--, 20-30% increase/decrease over the range; +++/---, 30-40% increase/decrease over the range; ++++/----, > 40% increase/decrease over the range.
